# Pulsatilla Powder Ameliorates Damp-Heat Diarrhea in Piglets Through the Regulation of Intestinal Mucosal Barrier and the Pentose Phosphate Pathway Involving G6PD and NOX

**DOI:** 10.3390/vetsci12050403

**Published:** 2025-04-25

**Authors:** Yunqi Qu, Qi Ma, Chenying Wang, Lifang Zhang, Haolian Feng, Siyue Lai

**Affiliations:** 1 Department of Traditional Chinese Veterinary Medicine, College of Veterinary Medicine, Southwest University, Chongqing 402460, China; kx31351@163.com (Y.Q.); xwsdh111111@163.com (C.W.); f1986199412@163.com (L.Z.); fhl19892004@outlook.com (H.F.); 19562220194@163.com (S.L.); 2 Institute of Traditional Chinese Veterinary Medicine, Southwest University, Chongqing 402460, China

**Keywords:** damp-heat diarrhea, pulsatilla powder, pentose phosphate pathway, glucose-6-phosphate dehydrogenase, NADPH oxidase, reactive oxygen species

## Abstract

Damp-heat diarrhea (DHD) in piglets presents as diarrhea and intestinal bleeding, significantly affecting both piglet health and the pig industry. Pulsatilla powder (PP), a herbal formulation composed of Pulsatilla, Rhizoma Coptidis, Phellodendron Bark, and Fraxini Cortex, has proven to be an effective treatment for DHD. Although the pentose phosphate pathway (PPP) has been associated with its therapeutic effects, the exact mechanism of action remains unclear. In this study, the DHD model in piglets was established to evaluate clinical symptoms, organ index, serum index, histological changes, colonic metabolites, and molecular mechanisms using techniques such as QPCR, ELISA, WB and metabolomics. PP improved intestinal health by restoring spleen and lung index, increasing LDL-C and HDL-C levels (HDL-C: *p* < 0.05), decreasing mRNA expression levels of *IFN-γ* and *TNF-α* mRNA (*p* < 0.01), and increasing *MUC1* and *MUC2* expression. Metabolomics analysis has identified 44 pathways, including pentose phosphate and glutathione pathways, and 132 differential metabolites have involved in DHD treatment. PP significantly reduced *G6PD* (*p* < 0.01), inhibited the pentose phosphate pathway, reduced NOX production (*p* < 0.01), and suppressed ROS production (*p* < 0.01). These effects alleviated oxidative stress and intestinal damage, demonstrating PP’s effectiveness in treating DHD by targeting critical enzymes and ROS levels.

## Simple Summary

Damp-heat diarrhea (DHD) is a prevalent condition in piglets, characterized by a high morbidity rate. It is primarily caused by elevated temperature and humidity, which pose significant challenges to the swine industry. Pulsatilla powder (PP), a herbal formulation composed of Pulsatilla, Rhizoma Coptidis, Phellodendron Bark, and Fraxini Cortex, has demonstrated promising efficacy in treating this condition. Previous studies have identified the pentose phosphate pathway (PPP) as a critical therapeutic target. Utilizing a piglet model induced by a high-sugar and high-fat diet, improper feeding practices, elevated temperature and humidity, and Escherichia coli, this study validated the role of the PPP and explored its key enzymes and metabolites. The results indicated that PP improved the morphology of the colonic mucosa by regulating colonic metabolites, reduced inflammation by modulating inflammation-related genes, and effectively treated DHD by downregulating the PPP enzymes glucose-6-phosphate dehydrogenase (G6PD), nicotinamide adenine dinucleotide phosphate oxidase (NOX1, NOX2, NOX4), and levels of reactive oxygen species (ROS).

## 1. Introduction

In recent years, piglet diarrhea has been one of the most prevalent and significant health challenges affecting piglets, particularly weaned piglets [1]. The condition is characterized by high incidence and mortality rates, significantly impacting the swine industry [2]. Post-weaning diarrhea (PWD) is a common issue in animal husbandry, often triggered by environmental and microbial stressors associated with the weaning process. These stressors disrupt the intestinal barrier, leading to clinical manifestations such as diarrhea, vomiting, intestinal bleeding, growth retardation, and other clinical adverse symptoms. In traditional Chinese veterinary medicine (TCVM), diarrhea is categorized as a disorder marked by frequent defecation, loose stools, or watery stools. The underlying pathophysiology of diarrhea is often attributed to spleen and stomach dysfunction, impairing digestion and the separation of clear and turbid substances, ultimately resulting in diarrhea. TCVM classifies diarrhea into several types based on clinical manifestations, including cold-damp diarrhea, damp-heat diarrhea, food-induced diarrhea, and diarrhea due to spleen deficiency, among others [1].

In southwest China and the Yangtze River basin, in the summer and autumn, piglets are often in high temperature and humidity environments and are susceptible to the influence of dampness and heat, which can trigger diarrhea due to damp-heat diarrhea (DHD) [3]. Clinically, piglets with DHD often present with elevated body temperature, increased thirst, abdominal discomfort, and yellowish-brown, watery feces, potentially mixed with pus and blood, accompanied by a foul odor, as well as scanty and dark red urine. These symptoms may arise from external damp-heat infections or dietary imbalances. From the perspective of modern veterinary medicine, high temperatures, humid environments exacerbate the condition by weakening the piglets’ immune resistance, rendering them more susceptible to pathogenic bacterial infections following dietary and environmental changes associated with weaning [4].

The integrity of the intestinal mucosa is closely linked to the redox balance within the gut [5]. Studies have shown that during weaning, piglets experience disruptions in redox homeostasis, leading to increased oxidase activity, elevated levels of free radicals, and the accumulation of peroxide products [6,7]. This imbalance contributes to oxidative damage [8,9], which includes cellular DNA damage, tissue injury, and stress responses [10]. The pentose phosphate pathway (PPP) in glucose metabolism plays a pivotal role in maintaining intestinal redox balance [11]. Enhanced activity of the PPP’s key enzyme, glucose-6-phosphate dehydrogenase (G6PD), drives the production of pentose phosphates and nicotinamide adenine dinucleotide phosphate (NADPH) [12]. While moderate levels of NADPH are essential for cellular function, excessive accumulation can activate NADPH oxidase (NOX), particularly its intestinal-related subtypes NOX1, NOX2, and NOX4 [13], leading to the overproduction of reactive oxygen species (ROS) [14]. Excessive ROS generation surpasses the organism’s antioxidant capacity, resulting in oxidative damage to intracellular DNA, proteins, and lipids. This ultimately leads to cellular and further intestinal damage. The glutathione cycle plays a critical role in counteracting this damage by reducing peroxides, scavenging excess ROS, and preventing further cellular injury, thereby maintaining intestinal health [15].

Pulsatilla powder (PP), a traditional formulation in TCVM, has been widely utilized to manage DHD [16]. It comprises four key herbal ingredients: *Pulsatilla*, *Rhizoma Coptidis*, *Phellodendron Bark*, and *Fraxini Cortexi*. PP exhibits significant antibacterial properties and is frequently employed to treat gastrointestinal disorders associated with diarrhea [17,18]. Previous studies conducted by our team have systematically demonstrated the efficacy of PP in alleviating symptoms of DHD in piglet models, including diarrhea, bloody stools, and mucus discharge [7]. Furthermore, metabolomic analyses have revealed that pulsatilla decoction modulates urinary metabolic markers associated with the PPP and the glutathione pathway, providing insights into its therapeutic mechanisms [7]. However, the specific pathway through which PP ameliorates colonic injury in DHD-affected piglets, particularly its regulation of PPP-related metabolites, remains unclear.

This study aims to investigate the effects of PP on the enzymes, genes, and proteins associated with the PPP in piglets with DHD. Using a DHD piglet model, we will evaluate the impact of PP on PPP-related metabolites from a metabolomic perspective. Additionally, the study will assess the effects of PP on colonic tissue morphology and inflammation to elucidate its therapeutic mechanisms. These findings aim at preventing robust experimental evidence and a theoretical foundation for future strategies to prevent and treating diarrhea in piglets.

## 2. Materials and Method

### 2.1. Animals

Eighteen piglets (Landrace × Meishan) were donated by the College of Animal Science at Southwest University. They were raised in the laboratory of the College of Veterinary Medicine at Southwest University using a traditional two-month weaning model until they reached eight weeks of age, with body weights ranging from 14 to 20 kg [19].

Piglets were acclimatized for 7 days prior to modeling, receiving a diet comprising 3% to 5% of their body weight (3 times/day), watered ad libitum, and were maintained at a temperature range of 20 to 24 °C. All procedures in this study adhered to the ethical standards set forth by Southwest University for animal experimentation (Approval No. IACUC-20240202-11) and were conducted in accordance with the nursery’s established protocols.

### 2.2. Preparation of PP

The pulsatilla powder (PP) was manufactured using the proportions specified in the Chinese Veterinary Pharmacopoeia (2020). *Pulsatilla* (SUTCM-20230701), *fraxini cortex* (SUTCM-20230702), *rhizoma coptis* (SUTCM-20230703), and *phellodendron bark* (SUTCM-20230704) were blended at a 4:4:2:3 ratio. All were obtained from Yongsheng Pharmacy in Rongchang, Chongqing, China, and authenticated by Prof. Cao Liting of the College of Veterinary Medicine at Southwest University. Shimadzu LC-20 AT HPLC System equipped with SPD-20AD UV detector is used to analyze four chemical components in PP. The fingerprints of PP samples are obtained by eluting compounds on Agilent ZORBAX SB-C18 column (5 μm, 150 nm × 4.6 nm, Santa Clara, CA, USA). The herbs were pulverized in a drug grinder and passed through screen No. 3 (The particle size is 0.355 mm ± 0.013 mm), and the powder was fully blended to produce PP, with 45 g per day administered to each piglet.

### 2.3. Primary Instruments and Reagents

Esculin (CAS: 531-75-9), esculetin (CAS: 305-01-1), palmatine hydrochloride (CAS: 10605-02-4), berberine hydrochloride (CAS: 633-65-8) were purchased from Nanjing Yuanzhi Biotechnology Co., Ltd. (Nanjing, China) Honey (GB14963) [20] was purchased from Jiangxi Huamao Health Products Development Co., Ltd. (Fuzhou, China). Lard was obtained from Shuikousi Market in Rongchang, Chongqing. 56° Red Star Erguotou liquor (GB/T 10781.2) [21] was sourced from Beijing Red Star Co., Ltd. (Beijing, China). The strain of Escherichia coli used in this study was isolated and identified by the College of Veterinary Medicine, Southwest University. Hematoxylin and eosin staining solution (batch no. 20220610) was purchased from Hunan Bicman Biotechnology Co., Ltd. (Changde, China). The PAS staining kit (Servicebio, G1008) was obtained from Shanghai Yuanju Biotechnology Center (Shanghai, China). Fully automated MicroplateReader (A-5082): Tecan Austria GmbH from Tecan (Shanghai) Laboratory Equipment Co., Ltd. (Shanghai, China); The porcine glucose-6-phosphate dehydrogenase (G6PP) ELISA kit (YJ955248) and the porcine reactive oxygen species (ROS) ELISA kit (YJ930026) were purchased from Shanghai Enlink Biotechnology Co., Ltd. (Shanghai, China). PrimeScript™ RT Master Mix (RR036A) and TB Green Premix Ex Taq™ II (RR820A) were sourced from Takara Bio (Beijing) Co., Ltd. (Beijing, China). The rotary microtome (model KD1508A) was purchased from Jinhua Kedi Instrument Co., Ltd. (Jinhua, China). The ultra-high sensitivity chemiluminescence imaging system was obtained from Beijing Wuzhou Oriental Technology Development Co., Ltd. (Beijing, China). β-actin antibody (bs-0061R) and NOX2 antibody (bs-3889R) were purchased from Beijing Boasen Biotechnology Co., Ltd. (Beijing, China). The NOX4 antibody (C14347-1-AP) was sourced from Wuhan Sanying Biotechnology Co., Ltd. (Wuhan, China). The DAB horseradish peroxidase color development kit was purchased from Shanghai Biyun Tian Biotechnology Co., Ltd. (Shanghai, China). The real-time quantitative PCR instrument (Archimed X6) was obtained from Kunpeng Gene Technology Co., Ltd. (Beijing, China). The low-density lipoprotein cholesterol (LDL-C) assay kit (A113-1-1) and high-density lipoprotein cholesterol (HDL-C) assay kit (A112-1-1) were purchased from Nanjing Jiancheng Bioengineering Institute (Nanjing, China).

### 2.4. Animal Model Preparetion and PP Treatment

**Figure 1:**
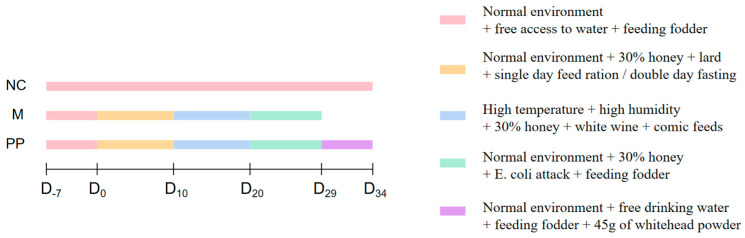
Modeling Process of Damp-Heat Diarrhea.

The experimental piglets were weaned at 8 weeks of age and provided with commercial feed and ad libitum access to water. Each piglet was weighed one day before the experiment, and 18 piglets were stratified by weight and randomized into three groups: the normal control group (NC), the model group (M), and the pulsatilla powder group (PP), with an average weight of 18.43 kg, and the model of DHD piglets was constructed with reference to previous experiments of our team [3]. Figure 1 depicts the modeling method: except for the NC group, the DHD piglets were modeled in three phases: high-sugar and high-fat phase, high heat and high humidity phase, and *E. coli* attack phase, and the piglets in the M and PP groups were fed 30% of the feed amount of honey per day. The high-sugar and high-fat period lasted from the first to the tenth day, and each piglet received 140 mL/1 kg body weight of lard on odd-numbered days of mixed feeding while fasting without water on even-numbered days. From 11 to 20 days, the piglets in the M and PP groups were placed in a self-constructed high-temperature and high-humidity shed (34 ± 1 °C, 94 ± 1%) for 8 h per day and given 70 mL/1 kg body weight of white wine. From 21 to 28 days, the piglets in the M and PP groups were gavaged with 30 mL of *E. coli* at a concentration of 2.75 × 10^11^ CFU/mL per day, while the piglets in the NC group were given commercial dietary feed and water ad libitum. After applying the DHD model, the piglets in the PP group were treated from the 30th to the 34th day. Forty-five grams of pulsatilla powder were mixed with an appropriate amount of feed. The mixture was moistened with warm water to achieve a slightly damp consistency without dust formation. Each piglet was then fed individually with a spoon, to ensure consistent drug intake.

### 2.5. Clinical Evaluation of DHD

During the test period, the activity, fecal condition, and nutritional hunger of piglets in each group were monitored. After the test, tissues from the spleen and lung were taken and weighed to determine the organ index of each group of piglets [Organ weight (kg)/Piglet weight (kg)]. The 5-Point Fecal Consistency Scale method was used: normal stool, dry, formed pellets with no residue or mucus (1 point); mildly soft stool, formed but with a wet surface, slightly adhering to the cage (2 points); moderately soft stool, loose and unformed, with significant water exudation (3 points); heavily pasty stool, with little to no solids, easily adhering to the anus or hair (4 points); watery stool, completely liquid, may contain blood or undigested food debris (5 points). The specific scores for each group are shown. Diarrhea was determined when feces were greater than or equal to three points, and the diarrhea index was calculated according to the formula.

$$\mathrm{Diarrhea}\;\mathrm{index}=\frac{\sum \mathrm{fecal}\;\mathrm{score}\times \mathrm{diarrhea}\;\mathrm{times}\;}{\mathrm{total}\;\mathrm{observation}\;\mathrm{times}}$$

After collecting blood, the serum was separated. The levels of LDH-C and HDL-C in the serum were detected using a kit and MicroplateReader. The colon tissue was excised and preserved in 4% neutral formaldehyde for the histological analysis. The remaining colon tissue was maintained at −80 °C as a spare.

### 2.6. Histopathological Examination

For histopathological examination, approximately 5 cm-long sections of colon tissue from each intestinal segment were fixed in 10% neutral buffered formalin for 24 h, preserved in 70% ethanol, embedded in paraffin, and sectioned into 5 μm thick slices. The slices were spread at 40 °C in a water bath, baked at 37 °C overnight, deparaffinized with xylene, and rehydrated using a gradient of 100% to 50% ethanol. Then stained with hematoxylin and eosin and dehydrated with a gradient of 50% to 100% ethanol. Another piece of tissue was taken, deparaffinized, and subjected to periodate staining, followed by schiff staining, hematoxylin staining, differentiation, blueing with a blueing solution, transparency with xylene, and sealing with neutral gum. Interpretation of the results revealed that glycogen and cup cells appeared purple-red, while the nuclei were pale blue.

### 2.7. Colon Metabolomics Profiling

#### 2.7.1. Preparation of Colon Tissue Samples

Samples were weighed before the extraction of metabolites. The samples were lyophilized and ground in a 2 mL Eppendorf tube containing a 5 mm tungsten bead for 1 min at 65 Hz in a Grinding Mill. Metabolites were extracted using 1 mL precooled mixtures of methanol, acetonitrile and water (*v*/*v*/*v*, 2:2:1) and then placed for 1 h ultrasonic shaking in ice baths. Subsequently, the mixture was placed at −20 °C for 1 h and centrifuged at 14,000× *g* for 20 min at 4 °C. The supernatants were recovered and concentrated to drying in a vacuum.

#### 2.7.2. UHPLC-MS/MS Analysis

Metabolomics profiling was analyzed using a UPLC-ESI-Q-Orbitrap-MS system (UHPLC, Shimadzu Nexera X2 LC-30AD, Shimadzu, Kyoto, Japan) coupled with Q-Exactive Plus (Thermo Scientific, San Jose, CA, USA).

For liquid chromatography (LC) separation, samples were analyzed using an ACQUITY UPLC^®^ HSS T3 column (2.1 × 100 mm, 1.8 μm) (Waters, Milford, MA, USA). The flow rate was 0.3 mL/min, and the mobile phase contained A: 0.1% FA in water and B: 100% acetonitrile (ACN). The gradient was 0% buffer B for 2 min and was linearly increased to 48% in 4 min, and then up to 100% in 4 min and maintained for 2 min, and then decreased to 0% buffer B in 0.1 min, with 3 min re-equilibration period employed.

The electrospray ionization (ESI) with positive mode and negative mode were applied for MS data acquisition separately. The HESI source conditions were set as follows: Spray Voltage: 3.8 kv (positive) and 3.2 kv (negative); Capillary Temperature: 320 °C; Sheath Gas (nitrogen) flow: 30 arb (arbitrary units); Aux Gas flow: 5 arb; Probe Heater Temp: 350 °C; S-Lens RF Level: 50. The instrument was set to acquire over the m/z range 70-1050 Da for full MS. The full MS scans were acquired at a resolution of 70,000 at m/z 200 and 17,500 at m/z 200 for MS/MS scans. The maximum injection time was set to 100 ms for MS and 50 ms for MS/MS. The isolation window for MS2 was set to 2 m/z, and the normalized collision energy (stepped) was set as 20, 30 and 40 for fragmentation.

#### 2.7.3. Data Processing and Analysis in Metabolomics

MS-DIAL analyzed raw colon metabolomics data, which included peak alignment, retention time correction, and peak area extraction. Metabolite structures were identified employing high-precision mass (mass tolerance < 10 ppm) and MS/MS data (mass tolerance < 0.02 Da). Public databases, including HMDB and MassBank, as well as BAYPRO Bio’s metabolite standard libraries, were searched for matches. Univariate statistical analysis was performed on the model, followed by multivariate statistical analysis using principal component analysis (PCA), partial least squares discriminant analysis (PLS-DA), and orthogonal partial least squares-discriminant analysis (OPLS-DA). The differential metabolites were identified and subjected to Venny analysis to identify shared potential differential metabolites based on variable importance in the projection (VIP) > 1 of the first principal component of the OPLS-DA model, as well as univariate statistical analysis with *p* value < 0.05 versus (FC ≥ 1.5 or FC ≤ 1/1.5). Following the basic data analysis, imported the screened differential metabolites into the Metaboanalyst database and used the Kyoto Encyclopedia of Genes and Genomes (KEGG) Pathway database (http://www.keggjp/keggpathway.html (accessed on 10 June 2024) to perform KEGG annotation. To identify the main pathways that have the strongest link with metabolite differences (impact > 0.1), the Metaboanalyst database (https://www.metaboanalyst.ca/ accessed on 15 June 2024)) was used for pathway enrichment analysis and topology analysis. The one-way ANOVA test was used to compare the groups, and differences were deemed statistically significant when *p* < 0.05 or −lg *p* > 0.1.

### 2.8. Quantitative Real-Time PCR

As directed by the TaKaRa Prime Script TM RT Master Mix kit, the TRIzol reagent was used to extract the total RNA from the frozen colon tissue samples, the RNA concentration was calculated, and the RNA was then inverted to cDNA and frozen at −20 °C. SuperReal PreMix Plus (SYBR Green) and the QuantStudio™ 7 Flex Real-Time Fluorescence Quantitative PCR System were used to conduct the qPCR experiment. Rox Reference Dye (50×) 0.2 μL, enzyme-free sterile water 3 μL, DNA template 1 μL, TBGreen Premix ExTaqI (2×) 5 μL, 0.4 μL each of upstream and downstream primers, and GAPPH served as the internal reference gene in the RT-qPCR reaction system. Pre-denaturation at 95 °C for 30 s, 95 °C for 5 s, and 60 °C for 34 s are the reaction conditions. Following 40 reaction cycles, the reaction was prolonged for one minute at 60 °C and melted for fifteen seconds at 95 °C. The following were the conditions of the reaction. The 2^−ΔΔCt^ technique was used to calculate the results. The primer sequences utilized for qPCR are presented in Table 1.

#### Determination of Colon G6PP and ROS

A centrifuge tube containing 20 mg of frozen colon tissue at −80 °C was filled with 1 mL of RIPA lysate buffer, along with 10 μL of phosphatase inhibitor, and 10 μL of protease inhibitor. The tissue was then homogenized using a tissue homogenizer. After lysing the tissue upper ice for 30 min, the top layer of the clear supernatant was collected as the sample and centrifuged in a refrigerated centrifuge at 4 °C. The BCA assay were strictly adhered to while measuring the protein concentration, and the results were used to standardize the protein concentration. An ELISA kit was employed to quantify the levels of G6PP and ROS was used to measure the G6PD and ROS content.

### 2.9. Western Blot Analysis

The protein concentration was measured using the BCA kit, followed by homogenization and denaturation at 100 °C for 10 min. The samples were then centrifuged at 4 °C and stored in a −80 °C freezer. Protein from the piglet colon was isolated using Tris-glycine gels, employing 1.5 mm 15-well plates with a 10% separating gel and a 5% stacking gel. The proteins were subsequently transferred to a polyvinylidene fluoride (PVDF) membrane. The membranes were blocked with a quick sealing solution for 15 min at room temperature. Primary antibodies were diluted in TBST at the following ratios: 1:500 for NOX2, 1: 500 for NOX4, and 1:1000 for GAPDH. The membrane was incubated with the primary antibody for 90 min at room temperature, followed by five washes with TBST. After washing, the membrane was incubated with the secondary antibody for an additional 90 min at room temperature. The GAPDH antibody served as a loading control. Finally, the bands were analyzed using the ImageJ (java 1.8.0-345) software.

### 2.10. Immunohistochemistry

The tissue samples collected from the inoculated piglet colon were preserved in formaldehyde. After paraffin embedding, the tissues were sectioned into 5 μm thick slices. The sections were heated in a sodium citrate antigen retrieval solution, deparaffinized, rehydrated, and treated with 5% hydrogen peroxide to eliminate endogenous peroxidase activity. To prevent nonspecific binding, 3% bovine serum albumin (BSA) was used. The primary antibodies were diluted in phosphate-buffered saline (PBS) at the following ratios: 1:300 for NOX2 and 1:300 for NOX4. Subsequently, the sections were incubated overnight at 4 °C with the primary antibodies. After the initial incubation, the sections were rinsed with PBS for five minutes and then incubated with a secondary antibody for three hours at 37 °C. The DAB horseradish peroxidase color development kit was meticulously followed for color development. Hematoxylin staining, dehydration, and resin sealing were then applied to the sections. Data analysis was performed using GraphPad Prism 8.0 and ImageJ.

### 2.11. Statistical Analysis

At least three biological replicates were conducted for each independent experiment. A *p*-value of <0.05 was considered statistically significant. GraphPad Prism 9.5.0 software was utilized to analyze the experimental data. Differences between groups were assessed using one-way ANOVA, with *p* < 0.05 indicating a significant difference and *p* < 0.01 indicating a highly significant difference.

## 3. Results

### 3.1. Identification of the Main Components in PP and PP Alleviated the Symptoms in DHD Piglets

**Figure 2:**
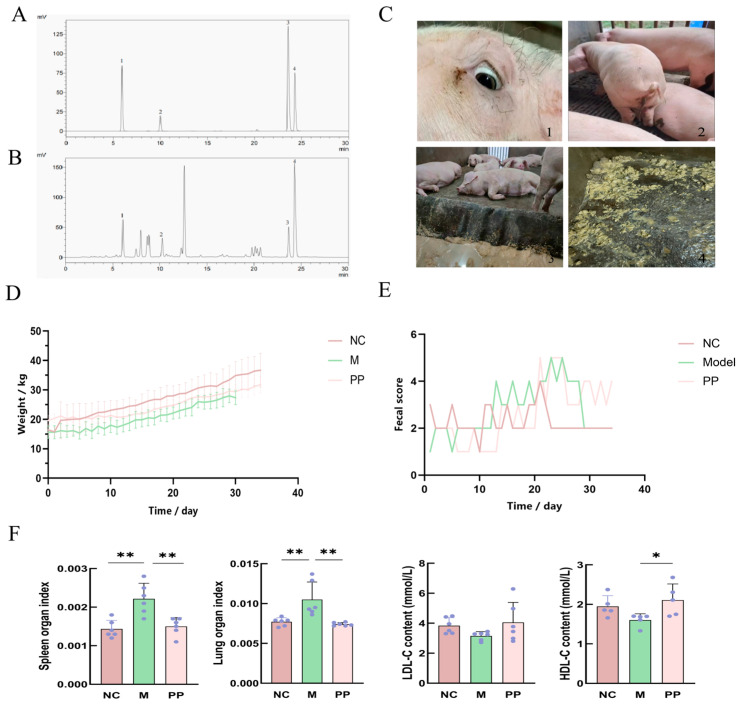
Ldentification of the main components in PP and PP alleviated the symptoms in DHD pigletsHPLC asay of PD sater, Chromatograms of Amixed standard at 335 mm (1 esculin, 2 esculetin,3 palmatine hydrochloride, 4 berberine hydrochloride) (**A**). HPLC curve chromatogram of B mixed standard at 335 nm (1 esculin, esculetin, 3 palmatine hydrochloride, 4 berberine hvdrochloride) (**B**); The clinical symptoms of piglets (**C**); Graph of piglet weigt change (**D**); Piglet fecal Score Chart (**E**); Biochemical indices (HD,DL) measurements and organ (spleen, lung) indices (**F**) (*p* < 0.05 * *p* < 0.01 **).

Figure 2A,B display the HPLC curves for both the standard and PP samples. The contents of esculin, esculetin, palmatine hydrochloride, berberine hydrochloride in PP were 5.676 ± 0.055 mg/g, 29.527 ± 0.036 mg/g, 2.250 ± 0.013 mg/g, 9.506 ± 0.196 mg/g, respectively. These components may be active ingredients involved in PP treatment of DHD.

Piglets in the model group (M) exhibited symptoms of DHD, including lethargy, decreased activity, loss of appetite, loose or watery feces, yellowish feces (Figure 2(C2,C4)), a fishy odor, and back edema around the eyes. As illustrated in Figure 2D, the weight of piglets in group M consistently increased throughout the modeling process. During the initial 10 days of high sugar and fat intake, the weights of piglets in both the M and PP groups exhibited fluctuations due to the feeding regimen; weights increased on feeding days and decreased on fasting days, resulting in a wavy pattern. From days 11 to 20, characterized by high temperature and humidity, the body weight of piglets in groups M and PP continued to rise. In the challenge stage from days 21 to 29, the weight of piglets in both groups M and PP increased at a slower rate. By the end of the modeling period, the average weight of piglets in the NC group was 33.4 kg, while the M group averaged 27.96 kg, and the PP group averaged 29.6 kg. During the treatment stage from days 30 to 34, the weight of piglets in the PP group gradually increased. The fecal score is shown in the figure, and the diarrhea index is calculated according to the numerical value. The diarrhea index was 28/34 = 0.82 in NC group, 60/29 = 2.07 in M group and 63/34 = 1.85 in PP group. The diarrhea index was 28/34 = 0.82 in NC group, 60/29 = 2.07 in M group and 63/34 = 1.85 in PP group. Biochemical results revealed reduced LDL-C and HDL-C levels in the M group compared to the NC group, though these differences, were not statistically significant. However, the spleen and lung indices in the M group were significantly higher than in the NC group *(p* < 0.01 and *p* < 0.05, respectively). Following PP treatment, these organ indices returned to near-normal levels, with a significant increase in HDL-C (*p* < 0.05). Although LDL-C levels increased in the PP group, the difference remained statistically insignificant.

### 3.2. PP Attenuated Colon Pathological Injury in DHD Piglets

**Figure 3:**
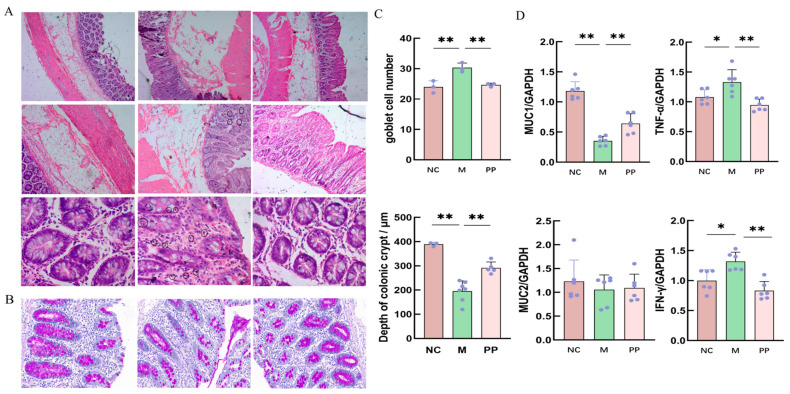
PP attenuated colon pathological injury in DHD piglets. Paraffin Sections of Colonic Tissue from Piglets with Damp-Heat Diarrhea (**A**); PAS (Periodic Acid-Schiff) Stained Sections of Colonic Tissue from Piglets with Damp-Heat Diarrhea (**B**); Measurement of Colonic Crypt Depth and Enumeration of Goblet Cells per Unit Area in Piglets with Damp-Heat Diarrhea (**C**); Quantification of Inflammatory Cytokines and Mucin Expression Levels in the Colonic Tissue of Piglets with Damp-Heat Diarrhea (**D**) (*p* < 0.05 * *p* < 0.01 **).

Histological analysis using H&E staining revealed that the colon tissues of the NC group exhibited well-defined villi structures and normal intestinal glands, with no signs of inflammatory infiltration (Figure 3A–C). In contrast, tissues in the M group showed mucosal thickening, crypt atrophy, goblet cell proliferation, immune cell infiltration, and red blood cell leakage. The PP group demonstrated significant improvements, presenting a mucosal structure that was nearly normal.

Quantitative PCR showed that inflammatory factors *IFN-γ* and *TNF-α* mRNA levels were significantly higher in the M group compared to the NC group (*p* < 0.05), while surface mucin *MUC1* levels were significantly lower (*p* < 0.01), and *MUC2* levels were lower (Figure 3D). PP treatment significantly reduced *IFN-γ* and *TNF-α* levels (*p* < 0.01), and increased *MUC1* levels (*p* < 0.01), and restored *MUC2* expression.

### 3.3. Colon Metabolomics Results

**Figure 4:**
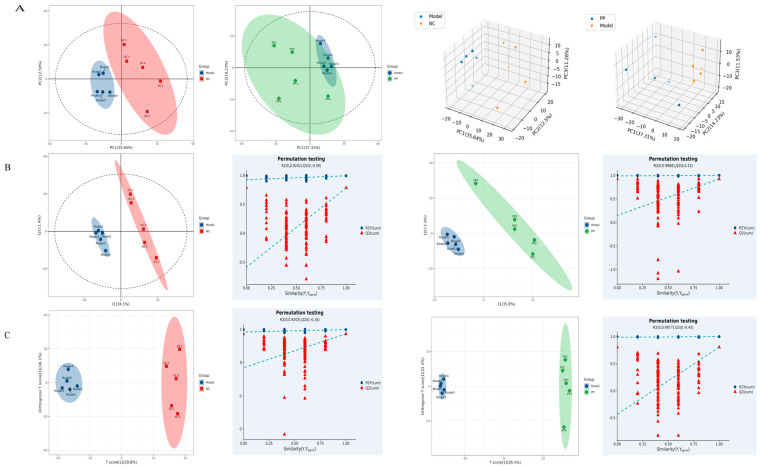
Colon metabolomics results1. Planar and 3D DAB (3,3′-Diaminobenzidine) Analysis Diagrams of Colonic Tissue from Piglets with Damp-Heat Diarrhea, Comparing NC vs. M, and M vs. PP Groups (**A**); Planar PLS-DA (Partial Least Squares Discriminant Analysis) and Model Validation Diagrams of Colonic Tissue from Piglets with Damp-Heat Diarrhea, Comparing NC vs. M, and M vs. PP Groups (**B**); Planar OPLS-DA (Orthogonal Partial Least Squares Discriminant Analysis) and Model Validation Diagrams of Colonic Tissue from Piglets with Damp-Heat Diarrhea, Comparing NC vs. M, and M vs. PP Groups (**C**).

The sample points of the NC group versus the M group and the M group versus the PP group were clearly separated, as can be seen in Figure 4A, which displays the multivariate statistical analysis PCA plots. Piglet colon sample points exhibited considerable separation and grouping in the NC group compared to the M group and M vs. PP, as seen by PLS-DA in Figure 4B. For the model validation, the NC group versus the M group had R2X = 0.459, R2Y = 0.992, and Q2 = 0.861, whereas the M group versus the PP group had R2X = 0.594, R2Y = 0.999, and Q2 = 0.923. Similarly, OPLS-DA colon tissue samples were used for analysis. Piglet colon sample sites in the NC group were significantly separated and clustered in comparison to the M group and M compared to PP, as seen in Figure 4C. R2X = 0.459, R2Y = 0.992, Q2 = 0.861 for the NC and M groups; R2X = 0.594, R2Y = 0.999, Q2 = 0.804 for the M and PP groups for model validation. The OPLS-DA model did not overfit, the experimental data were established for PLS-DA, the model was dependable, the DHD piglet model modeling was successful, and PP treatment of the DHD piglets was successful.

**Figure 5:**
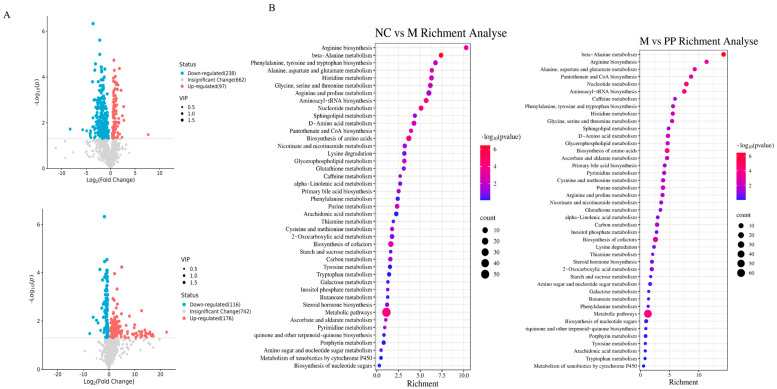
Colon metabolomics results2. Volcano Plots of Univariate Analysis for Colonic Tissue from Piglets with Damp-Heat Diarrhea, Comparing NC vs. M, and M vs. PP Groups (**A**); KEGG Enrichment Plots for Colonic Tissue from Piglets with Damp-Heat Diarrhea, Comparing NC vs. M, and M vs. PP Groups (**B**).

The volcano and heat maps were utilized to visualize the differential metabolite profiles of the screened compounds; red dots represented upregulated metabolites, while blue dots indicated downregulated metabolites (Figure 5A). Differential metabolite analysis identified 318 metabolites between the NC and M groups, and 273 metabolites between the M and PP groups. A total of 132 overlapping metabolites were identified, including D-gluconic acid, pyroglutamic acid, ornithine, and other compounds (For details, see File S1, Significantly Different Metabolites). Metabolic pathway analysis linked these 132 potentially distinct metabolites to 44 pathways (Figure 5B), with the glutathione and pentose phosphate pathways prominently implicated in the pathophysiology of DHD and the therapeutic effects of PP. For detailed information on differential metabolites and significant pathways, please refer to the Supplementary Material (For details, see File S2, Significant metabolic pathways).

### 3.4. Inhibitory Effect of PP on G6PD/ROS Signaling Pathway

**Figure 6:**
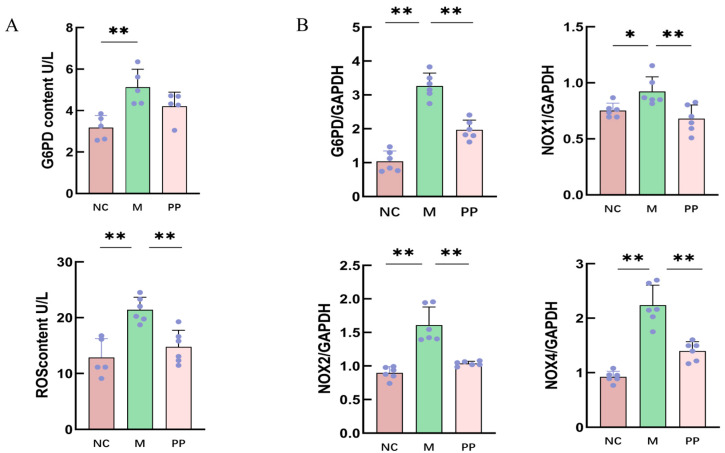
Elisa and RT-qPCR results. Elisa Measurement of G6PD and ROS Levels in Colonic Tissue from Piglets with Damp-Heat Diarrhea (**A**); RT-qPCR Analysis of *G6PD*, *NOX1*, *NOX2*, and *NOX4* mRNA Expression in Colonic Tissue from Piglets with Damp-Heat Diarrhea (**B**) (*p* < 0.05 * *p* < 0.01 **).

ELISA results showed elevated G6PD and ROS levels in the M group compared to the NC group (*p* < 0.01) (Figure 6A). These levels were significantly reduced in the PP group, suggesting PP’s inhibitory effect on oxidative stress in the colon.

#### 3.4.1. Gene Expression Analysis of Pentose Phosphate Pathway

qPCR revealed significantly higher expression of *G6PD*, *NOX1*, *NOX2*, and *NOX4* mRNA in the M group compared to the NC group (*p* < 0.01). PP treatment significantly downregulated these genes (Figure 6B), demonstrating its regulatory effects oncthe pentose phosphate pathway.

#### 3.4.2. Protein Expression of NOX2 and NOX4

**Figure 7:**
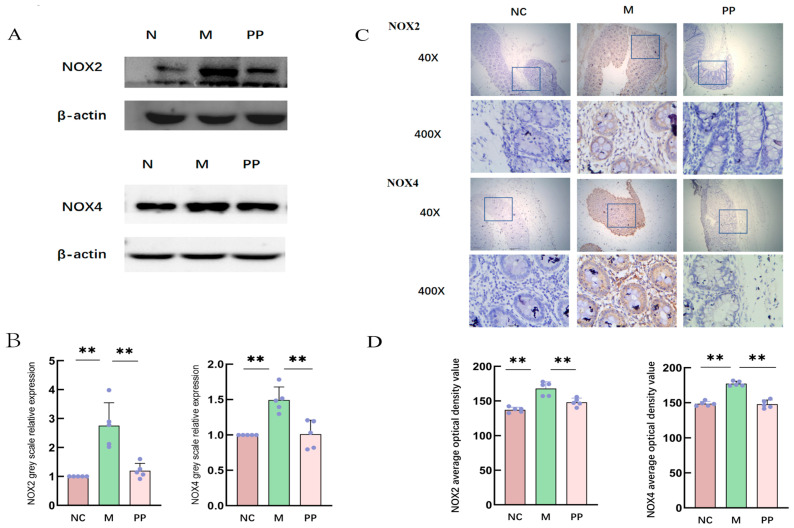
Protein expression of NOX2 and NOX4. Western Blot Analysis of NOX2 and NOX4 Protein Expression in Colonic Tissue from Piglets with Damp-Heat Diarrhea (**A**); Immunohistochemical Staining of NOX2 and NOX4 Proteins in Colonic Tissue from Piglets with Damp-Heat Diarrhea, Showing Localization and Expression Levels (**B**); Immunohistochemical determination of the protein locations of NOX2 and NOX4 in the colon tissues of piglets with damp-heat diarrhea (**C**); Immunohistochemistry was used to determine the relative expression levels of NOX2 and NOX4 proteins in the colon tissues of piglets with damp-heat diarrhea (**D**). (*p* < 0.01 **).

Western blot analysis showed elevated protein expression of NOX2 and NOX4in M group compared to the NC group, PP treatment significantly reduced the expression levels of both proteins (*p* < 0.01) (Figure 7A,B).

**Figure 8:**
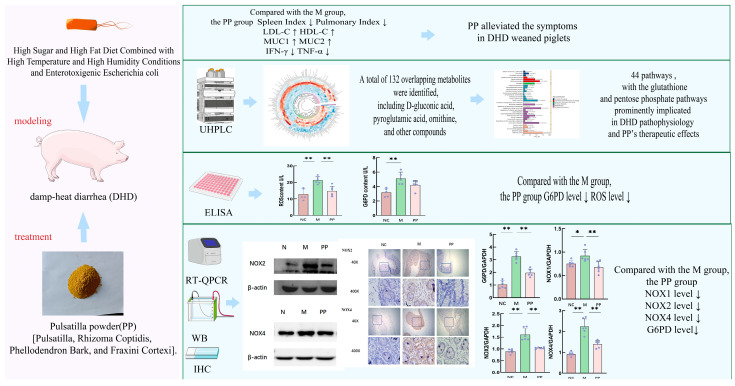
Integrated Representation of the Relationship between Metabolomics and Experimental Results (Some of the illustrations are from https://bioicons.com/, accessed on 10 June 2024). “↑” indicates an increase in its expression level. “↓” indicates a decrease in its expression level. (*p* < 0.05 * *p* < 0.01 **).

Immunohistochemical analysis showed NOX2 was primarily expressed in macrophages and neutrophils, while NOX4 was primarily expressed in inflammatory and mesenchymal cells, including neutrophils and lymphocytes; Each group selected five different sample results and analyzed the results with ImageJ software, the mean optical density value of NOX2 proteins in the M group was higher compared to the NC group, and the mean optical density value of NOX4 proteins was highly significant (*p* < 0.01). PP treatment significantly decreased the mean optical density values (*p* < 0.01). The experimental summary is shown in Figure 8.

## 4. Discussion

In this experiment, damp-heat diarrhea (DHD) in piglets is induced through a combination of high-sugar, high-fat, high-temperature, and high-humidity conditions, along with an infection by enterotoxigenic Escherichia coli (ETEC) [3]. DHD is characterized by an accumulation of damp heat in the intestines, where the majority of pathological changes are concentrated [22]. Maintaining the structural integrity of the intestinal mucosa is essential for mitigating the inflammatory response in gastrointestinal disorders such as inflammatory bowel disease [23]. Histopathological analyses of group M (DHD model) revealed crypt atrophy, distortion, goblet cell increased, erythrocyte and inflammatory cell infiltration, and disruption of tissue continuity. These findings are consistent with the known pathological features of ETEC-induced diarrhea and the traditional Chinese medicine (TCM) model of damp-heat diarrhea [3].

Diarrhea is often associated with a pronounced inflammatory response [24]. Key inflammatory mediators, particularly interferon-gamma (IFN-γ) and tumor necrosis factor-alpha (TNF-α), play pivotal roles in the progression of inflammation and tissue damage. TNF-α, a pro-inflammatory cytokine, can trigger the release of platelet-activating factors and reactive oxygen species (ROS), and exacerbate intestinal intestinal hypoxia and ischemia, which contribute to mucosal injury [25]. Moreover, TNF-α promotes the expression of additional cytokines, such as interleukin-1 beta (IL-1β), IL-6, IL-8, and IFN-γ, amplifying the inflammatory cascade [26]. IFN-γ binds to its receptor (IFN-γR1/2), activating Janus kinase (Jak) signaling pathways and transcriptional processes involved in immune modulation, lymphocyte activation, antigen presentation, cell proliferation, apoptosis, and antimicrobial responses. The elevated levels of TNF-α and IFN-γ observed in the colons of DHD-affected piglets in this study corroborate previous research [27,28].

MUC1 and MUC2, critical components of the intestinal barrier, are essential therapeutic targets for managing diarrhea [29]. MUC1 is often upregulated during enterocolitis and may contribute to epithelial proliferation and inflammation, while MUC2, secreted by goblet cells, is vital for maintaining the mucosal barrier and microbial homeostasis [30,31]. Reduced expression of MUC2 is linked to associated with an increased risk of intestinal inflammation, including colitis [32]. In the current study, the mRNA levels of MUC1 and MUC2 were significantly decrease in the colon tissue of the DHD model, indicating mucosal damage. Treatment with pulsatilla powder (PP) not only downregulated TNF-α and *IFN-γ* levels but also restored the expression of MUC1 and MUC2, facilitating mucosal repair and alleviating the symptoms of DHD.

In Chinese medicine theory, the ‘spleen’ is a comprehensive concept that encompasses digestion, nervous system function, immune response, endocrine activity, and other multi-system functions. It is responsible for regulating the functions of internal organs and tissues, facilitating nutrient absorption and energy conversion, maintaining homeostasis within the internal environment [33]. An internal environmental imbalance brought on by a weak spleen impairs intestinal mucosal immune function and intestinal flora balance. It also affects the body’s water metabolism, which leads to the buildup of pathogenic salt and water metabolites in the body. In TCM ethics, this then results in the development of excessive dampness, which can quickly cause damp-heat enteritis [34]. Overconsumption of fatty and sugary foods can harm the stomach and spleen, impairing their ability to digest and transport nutrients, which causes fluids to pool in the body and creates internal moisture. If the spleen function is already compromised, the body can easily retain heat and moisture from the outside when infections and a hot, humid environment infiltrate. At this stage, the interaction between exogenous and endogenous dampness and heat leads to the development of the damp-heat syndrome and initiates an ongoing pathological cycle of internal dampness creation. As the body’s largest immunological organ, the spleen plays a crucial role in controlling inflammation in modern medicine [24]. The systemic reaction to damp-heat diarrhea is reflected in changes to the splenic organ index. This index increases as the spleen produces antibodies to combat inflammation following an ETEC infection [29]. By reducing the synthesis of pro-inflammatory molecules, PP therapy mitigates this response and decreases the organ index, thereby alleviating the inflammatory burden on the spleen.

Lipid metabolism and inflammatory are closely interlinked, with acute inflammation disrupting intracellular lipid balance. Cholesterol, vital for cell membrane synthesis and rapid cellular proliferation, is transported in the form of LDL-C and HDL-C [35]. These lipoproteins are critical for cholesterol efflux and maintaining homeostasis [36]. Dysregulated LDL-C and HDL-C levels are associated with inflammation, as TNF-α and other inflammatory agents can disrupt cholesterol metabolism [37,38]. PP normalized lipid profiles in the DHD model, suggesting its role in maintaining cholesterol balance and cellular repair.

The pentose phosphate pathway (PPP) is closely associated with oxidative damage in the gut. As a key metabolic route for glucose-6-phosphate (G6PD), the PPP, also known as the hexose monophosphate shunt, generates reduced nicotinamide adenine dinucleotide phosphate (NADPH)and ribose-5-phosphate. NADPH plays a critical role as a reducing agent in many cell synthetic reactions, including fatty acid and sterol synthesis. However, excessive production of NADPH has been linked to tumor development [39].

NADPH oxidase (NOX) is a family of proteins that facilitate electron transfer across cellular membranes, generating reactive oxygen species (ROS) upon activation [40]. ROS, produced during aerobic metabolism, includes both free radicals and non-radical oxidants, such as peroxides and hydroxyl radicals [41,42]. While moderate levels of ROS support physiological processes, including cell growth, apoptosis, and the regulation of inflammatory responses [41,43], excessive ROS can overwhelm antioxidant defenses, leading to damage of DNA, proteins, and other cellular components [44]. Notably, NOX expression is elevated during intestinal inflammation [45]. Glutathione, a key antioxidant, plays a crucial role in mitigating ROS-induced cellular damage.

The pentose phosphate pathway (PPP) serves as the primary source of NADPH for NADPH oxidase (NOX)-mediated reactive oxygen species (ROS) production, with glucose-6-phosphate dehydrogenase (G6PD) acting as its rate-limiting enzyme [46]. In the DHD model, elevated levels of G6PD were consistent with previous findings [47]. PP mitigated intestinal damage by decreasing G6PD levels and ROS production. Furthermore, reducing the NADPH/NADP+ ratio has been shown to alleviate symptoms of enteritis [48].

Among the members of the NOX family, NOX1, NOX2, and NOX4 are particularly relevant to enteritis. NOX1, primarily located in colonic epithelial cells, supports the functions of goblet and absorptive cells, epithelial proliferation, and tissue repair under normal conditions [49,50]. However, excessive expression of NOX1 exacerbates inflammation. NOX2, which is typically inactive, is stimulated by external factors to produce reactive oxygen species (ROS), thereby amplifying inflammatory responses [51]. Additionally, ROS can activate NOX2 through intracellular signaling pathways, establishing a positive feedback loop [52]. The activation of NOX2, via proteolytic granules, enhances neutrophil bacteriostasis to counteract bacterial invasion [53]. Nevertheless, excessive activation of NOX2 can lead to oxidative stress, resulting in increased levels of oxygen free radicals, promoting apoptosis, and damaging the intestinal mucosal barrier [51]. Although NOX4 is predominantly expressed in the kidneys, it is also present in colonic epithelial cells, where it contributes to intestinal inflammation through oxidative stress. The upregulation of NOX4 plays a significant role in the pathogenesis of enteritis [54,55]. Silencing NOX4 has been shown to inhibit the metastasis of colon cancer [56].

In this study, PP inhibited the expression of NOX1, NOX2, and NOX4, thereby reducing oxidative stress and promoting mucosal regeneration. By modulating the NADPH/NADP+ ratio and regulating the rate-limiting enzyme of the pentose phosphate pathway, G6PD, PP decreased the production of reactive oxygen species (ROS) and mitigated intestinal damage. Its capacity to enhance intestinal antioxidant defenses highlights its therapeutic potential for treating damp-heat diarrhea.

## 5. Conclusions

This study employed a metabolomics-based approach to investigate the therapeutic mechanism of pulsatilla powder (PP) in treating damp-heat diarrhea in piglets. A total of 44 metabolic pathways, including the glutathione and pentose phosphate pathways, along with 132 metabolites, were identified as potential targets. PP treatment significantly downregulated key enzymes such as G6PD, NOX1, NOX2, and NOX4, leading to a reduction in reactive oxygen species (ROS) production and oxidative damage. Additionally, it restored the morphology of the colonic mucosa, regulated inflammatory markers, and improved lipid metabolism. These findings underscore the multi-target and multi-pathway effects of PP, providing a scientific basis for its clinical application in veterinary medicine and offering insights into its role in managing gastrointestinal disorders.

## Figures and Tables

**Table 1:** Modeling Process of Damp-Heat Diarrhea.

Primer Name	Sequences (5′→3′)
*GAPDH*-F	TCGGAGTGAACGGATTTGGC
*GAPDH*-R	TGACAAGCTTCCCGTTCTCC
*MUC1*-F	GTGCTTACAGGTGAGGGGC
*MUC1*-R	ACAGATCCTGGCCTGAACTT
*MUC2*-F	GAACGGGGCCATGGTCAG
*MUC2*-R	AGCATGACCGAGTCCTCTCT
*TNF-α*-F	GGCCCAAGGACTCAGATCAT
*TNF-α*-R	CTGTCCCTCGGCTTTGACAT
*IFN-γ*-F	TGAAGAATTGGAAAGAGGAGAGTGA
*IFN-γ*-R	GCTCCTTTGAATGGCCTGGT
*NOX1*-F	AATGGCATCCCTTTACCCTGACCT
*NOX1*-R	CTTGGAACTGGCGAATGCTGTTGT
*NOX2*-F	CCATATCCGCATTGTTGGCG
*NOX2*-R	CCGTCCACAGCGATCTTAGG
*NOX4*-F	GGAACGCACTACCAGGATGT
*NOX4*-R	CAGGTCTGCGGAAAGTTAGC

## Data Availability

All the data presented in the study are included in the article; further inquiries can be directed to the corresponding author.

## Supplementary Materials

The following supporting information can be downloaded at: https://www.mdpi.com/article/10.3390/vetsci12050403/s1, Supplementary File S1: Significantly Different Metabolites; Supplementary File S2: Significant Metabolic Pathways.
