# Supplementary material for: Pulsatilla Powder Ameliorates Damp-Heat Diarrhea in Piglets Through the Regulation of Intestinal Mucosal Barrier and the Pentose Phosphate Pathway Involving G6PD and NOX
Source: Vet Sci. 2025 Apr 25;12(5):403. doi: 10.3390/vetsci12050403 (PMC12116046; doi:10.3390/vetsci12050403)
Supplement: Supplementary file 1 [file vetsci-12-00403-s001.zip › Supplementary file 1, Significantly Different Metabolites.pdf]

Supplementary file 1, Significantly Different Metabolites

NO.	Alignment ID	Metabolite name	Rt(min)	Adduct type	NC vs M			M vs PP		
					P.value	FC	Significant	P.value	FC	Significant
1	NEG10480	Maltose	0.938	[M+Cl]-	0.0048	0.1392	↓	0.0005	8.6942	↑
2	NEG10763	S-Adenosylhomocysteine	5.216	[M-H]-	0.0002	2.2693	↑	0.0001	0.4150	↓
3	NEG11215	trans-4-[4-(4-Amino-7,7-dimethyl-7H-pyrimido[4,5-b][1,4]oxazin-6-yl)phenyl]cyclohexaneacetic acid	10.862	[M-H]-	0.0037	0.2861	↓	0.0288	7.6037	↑
4	NEG11443	Detca	11.726	[M-H]-	0.0009	0.3354	↓	0.0272	3.6669	↑
5	NEG11529	Citreoviridin	11.651	[M-H]-	0.0155	0.1839	↓	0.0260	14.4557	↑
6	NEG1172	N-lactoyl-Glycine	0.878	[M-H]-	0.0001	1.4863	↑	0.0002	0.6762	↓
7	NEG12848	Melledonol	9.987	[M-H]-	0.0064	0.3990	↓	0.0181	6.0700	↑
8	NEG12971	"N1,N10-Dicoumaroylspermidine"	9.481	[M-H]-	0.0326	0.1695	↓	0.0322	19.9758	↑
9	NEG13524	Coprocholic acid	13.072	[M-H]-	0.0160	0.2884	↓	0.0334	5.6480	↑
10	NEG1374	Patulin	8.333	[M-H]-	0.0035	0.6270	↓	0.0158	2.7975	↑
11	NEG1382	2,6-Dihydroxybenzoate	8.091	[M-H]-	0.0013	0.3148	↓	0.0143	3.7278	↑
12	NEG16169	Corosin	12.624	[M-H]-	0.0227	0.0584	↓	0.0492	181.8056	↑
13	NEG1770	Ethyl benzoate	9.814	[M-H]-	0.0105	0.6560	↓	0.0282	2.0190	↑
14	NEG18831	LPI(18:2)	13.368	[M-H]-	0.0261	1.7510	↑	0.0148	0.5046	↓
15	NEG19538	LPI(20:4)	13.374	[2M-H]-	0.0011	2.0849	↑	0.0032	0.4586	↓
16	NEG2067	Cyclamic acid	6.47	[M-H]-	0.0331	205.2895	↑	0.0332	0.0056	↓
17	NEG2284	2,4-Dimethylbenzenesulfonic acid	8.082	[M-H]-	0.0162	0.4348	↓	0.0192	876.5256	↑
18	NEG2369	Glycylleucine	5.997	[M-H]-	0.0269	1.7166	↑	0.0008	0.3698	↓

19	NEG237	Phosphoric acid	0.893	[M-H]-	0.0041	2.2078	↑	0.0230	0.6329	↓
20	NEG2410	N-Lactoylvaline	7.751	[M-H]-	0.0065	1.8716	↑	0.0019	0.3909	↓
21	NEG24558	Soyasaponin I	12.418	[M-H]-	0.0227	0.0334	↓	0.0051	28.2494	↑
22	NEG2597	(R)-3-Hydroxy-5-phenylpentanoic acid	11.289	[M-H2O-H]-	0.0181	0.1813	↓	0.0394	4.4202	↑
23	NEG2899	N-Lactoylleucine	9.152	[M-H]-	0.0017	2.0635	↑	0.0006	0.3736	↓
24	NEG3164	5-(3',5')-Dihydroxyphenyl-gamma-valerolactone	9.558	[M-H]-	0.0137	0.1793	↓	0.0126	8.2612	↑
25	NEG318	GABA	0.878	[M-H]-	0.0002	1.5071	↑	0.0008	0.6850	↓
26	NEG339	(S)-3-Hydroxybutyric acid	2.601	[M-H]-	0.0012	1.4612	↑	0.0014	0.6561	↓
27	NEG3480	2-Hydroxydecanedioic acid	9.701	[M-H]-	0.0118	0.4048	↓	0.0087	4.7357	↑
28	NEG3516	Pantothenic acid	6.192	[M-H]-	0.0278	1.4180	↑	0.0013	0.4831	↓
29	NEG3585	Methyl 5-hydroxyoxindole-3-acetate	9.611	[M-H2O-H]-	0.0111	0.2561	↓	0.0109	6.2330	↑
30	NEG3695	2-Amino-1-methyl-6-phenylimidazo(4,5-b)pyridine	9.468	[M-H]-	0.0030	0.3277	↓	0.0148	4.1919	↑
31	NEG3875	5-Heptyltetrahydro-2-oxo-3-furancarboxylic acid	10.439	[M-H]-	0.0014	0.3666	↓	0.0113	3.0645	↑
32	NEG4437	2-Carboxy-4-dodecanolide	10.195	[M-H]-	0.0167	0.3405	↓	0.0108	3.8243	↑
33	NEG4541	Polyethylene, oxidized	10.881	[M-H]-	0.0253	0.5161	↓	0.0486	2.4322	↑
34	NEG4657	3-Hydroxydodecanedioic acid	10.437	[M-H]-	0.0010	0.2242	↓	0.0105	5.2238	↑
35	NEG4839	2,6-Diaminopurine 2',3'-dideoxyriboside	10.435	[M-H]-	0.0001	0.2562	↓	0.0073	4.5089	↑
36	NEG4952	N-lactoyl-Tyrosine	7.775	[M-H]-	0.0238	1.8429	↑	0.0068	0.4060	↓
37	NEG4987	2-Hydroxy-4-Methoxychalcone	10.897	[M-H]-	0.0092	0.1287	↓	0.0427	3.5802	↑
38	NEG5001	4-Methylene-2-octyl-5-oxotetrahydrofuran-3-carboxylic acid	9.989	[M-H]-	0.0004	0.2745	↓	0.0104	2.8400	↑
39	NEG5146	3'-Hydroxyequol	11.351	[M-H]-	0.0257	0.3756	↓	0.0258	5.0726	↑
40	NEG5163	Tetradecanedioic acid	12.866	[M-H]-	0.0004	0.2042	↓	0.0155	5.0496	↑

41	NEG5568	Acoric acid	12.554	[M-H]-	0.0000	0.0829	↓	0.0257	6.2131	↑
42	NEG5876	Glutamyl-gamma-glutamate	0.965	[M-H]-	0.0142	1.9535	↑	0.0388	0.7316	↓
43	NEG6025	Isoleucylphenylalanine	9.11	[M-H]-	0.0311	0.3304	↓	0.0413	2.5158	↑
44	NEG6170	2'-O-Methylinosine	6.11	[M-H]-	0.0009	1.5798	↑	0.0025	0.5539	↓
45	NEG6243	Xanthosine	5.84	[M-H]-	0.0017	2.3542	↑	0.0002	0.3092	↓
46	NEG6422	Zaluzanin D	10.778	[M-H]-	0.0020	0.1610	↓	0.0162	8.1341	↑
47	NEG6801	2'-O-Methylguanosine	6.093	[M-H]-	0.0006	1.9079	↑	0.0122	0.5519	↓
48	NEG6836	7C-aglycone	12.166	[M-H]-	0.0002	0.3491	↓	0.0362	3.8030	↑
49	NEG6913	Farrerol	10.509	[M-H]-	0.0088	0.4372	↓	0.0484	2.3666	↑
50	NEG6923	desmethylclomipramine	11.732	[M-H]-	0.0268	0.3690	↓	0.0476	0.4925	↓
51	NEG7032	Enterodiol	11.086	[M-H]-	0.0128	0.4819	↓	0.0290	5.6997	↑
52	NEG7127	Aleuretic Acid	10.915	[M-H]-	0.0002	0.2911	↓	0.0353	3.7406	↑
53	NEG7369	N-Acetylneuraminic acid	0.919	[M-H]-	0.0145	1.7134	↑	0.0420	0.7391	↓
54	NEG7481	N2,N2-Dimethylguanosine	6.597	[M-H]-	0.0030	2.1998	↑	0.0161	0.5314	↓
55	NEG7637	2-Hydroxyenterolactone	11.091	[M-H]-	0.0021	0.1227	↓	0.0103	15.1599	↑
56	NEG7655	9,10-Epoxyoctadecanoic acid	13.577	[M-H]-	0.0258	0.3662	↓	0.0117	3.7607	↑
57	NEG7685	2-((2,6-Diethylphenyl)(methoxymethyl)amino)-2-oxo-ethanesulfonic acid	8.679	[M-H]-	0.0008	0.1775	↓	0.0106	10.1711	↑
58	NEG7754	Menthol-glucoronide	11.453	[M-H]-	0.0112	0.3212	↓	0.0217	4.6545	↑
59	NEG7827	Irigenol	1.153	[M-H]-	0.0435	5.0813	↑	0.0122	0.0527	↓
60	NEG792	Pyrroline hydroxycarboxylate	0.878	[M-H]-	0.0000	1.5860	↑	0.0001	0.6448	↓
61	NEG8333	(9R,10S,12Z)-9,10-Dihydroxy-8-Oxo-12-Octadecenoic Acid	10.891	[M-H]-	0.0381	0.5833	↓	0.0118	1.7951	↑
62	NEG8410	Pilosin	12.204	[M-H]-	0.0311	0.2509	↓	0.0364	5.7488	↑

63	NEG8411	3-(1,2-dihydroxypropyl)-1,6,8-trihydroxyanthracene-9,10-dione	8.452	[M-H]-	0.0271	0.2336	↓	0.0432	65.8673	↑
64	NEG849	Methylsuccinic acid	8.954	[M-H]-	0.0074	0.2238	↓	0.0139	3.2128	↑
65	NEG862	Aspartate	0.875	[M-H]-	0.0001	1.7030	↑	0.0001	0.6123	↓
66	NEG8833	D-myo-Inositol 1,4-bisphosphate	0.721	[M-H]-	0.0273	2.7742	↑	0.0070	0.2190	↓
67	NEG8911	Leu-Pro-Ile	9.172	[M-H]-	0.0094	2.1687	↑	0.0037	0.3364	↓
68	NEG9564	13,14-Dihydro PGF2a	11.995	[M-H]-	0.0000	0.2139	↓	0.0251	3.1335	↑
69	NEG9729	Prednisolone	12.346	[M-H]-	0.0022	0.1903	↓	0.0068	8.4501	↑
70	NEG986	Creosol	8.346	[M-H]-	0.0127	0.3920	↓	0.0197	13.0107	↑
71	NEG9918	11b,21-Dihydroxy-3,20-oxo-5b-pregnan-18-al	13.418	[M-H2O-H]-	0.0294	0.0540	↓	0.0252	29.6633	↑
72	POS10090	5,6-Dhet	13.163	[M+H]+	0.0376	0.0558	↓	0.0317	21.1853	↑
73	POS10204	Isoleucyl-Prolyl-Isoleucine	9.193	[M+H]+	0.0110	2.0035	↑	0.0068	0.3768	↓
74	POS1072	Isoindoline	5.714	[M+NH4]+	0.0034	1.4821	↑	0.0004	0.5386	↓
75	POS1090	2,3-Dihydrobenzofuran	2.903	[M+H]+	0.0284	0.1145	↓	0.0260	7.1890	↑
76	POS10986	Fosfructose	1.144	[M+Na]+	0.0450	2.6381	↑	0.0029	0.1447	↓
77	POS11058	Melibiose	1.39	[M+Na]+	0.0061	0.0817	↓	0.0020	9.7974	↑
78	POS1137	3-Aminopicolinaldehyde	2.25	[M+NH4]+	0.0028	1.5688	↑	0.0092	0.6067	↓
79	POS1171	Nicotinic acid	2.18	[M+H]+	0.0341	0.1701	↓	0.0414	3.1476	↑
80	POS12506	Droperidol	9.122	[M+Na]+	0.0151	0.0790	↓	0.0169	8.6113	↑
81	POS127	Tetrahydropyridine	1.15	[M+NH4]+	0.0060	1.4405	↑	0.0002	0.5842	↓
82	POS13021	3-Hydroxyhexadecanoylcarnitine	11.257	[M+CH3OH+H]+	0.0170	0.1087	↓	0.0115	5.5373	↑
83	POS1374	Pyroglutamic acid	1.34	[M+H]+	0.0004	1.5820	↑	0.0170	0.7331	↓
84	POS13860	Dicoumaroyl Spermidine	9.497	[M+H]+	0.0267	0.1563	↓	0.0299	17.9323	↑

85	POS13861	Lunarine	9.353	[M+H]+	0.0199	0.1026	↓	0.0301	12.2174	↑
86	POS1389	Pipecolic acid	1.151	[M+H]+	0.0172	1.3723	↑	0.0003	0.5882	↓
87	POS13911	Oleanolic acid	12.227	[M+H-H2O]+	0.0078	6.5153	↑	0.0205	0.3010	↓
88	POS13994	22-Hydoxy-2-hopen-1-one	13.379	[M+H]+	0.0124	0.1362	↓	0.0125	6.0839	↑
89	POS14726	2,2,2-trifluoroethyl 2-{N-[2-(5-methoxy-2-methylindol-3-yl)ethyl]carbamoyl}cyc lohexanecarboxylate	11.15	[M+Na]+	0.0282	0.0582	↓	0.0274	27.7228	↑
90	POS14757	Aspartyl adenylate	5.77	[M+H]+	0.0316	3.6564	↑	0.0303	0.2438	↓
91	POS1482	Dehydro-P-Cymene	9.036	[M+H]+	0.0129	0.4119	↓	0.0287	2.2373	↑
92	POS1544	Carveol	9.027	[M+H-H2O]+	0.0025	0.3583	↓	0.0130	2.9642	↑
93	POS166	Cyclopentylamine	2.362	[M+H]+	0.0005	1.4806	↑	0.0000	0.2849	↓
94	POS167	2-Methylpyrrolidine	2.102	[M+H]+	0.0008	0.3002	↓	0.0136	5.6589	↑
95	POS1684	Urocanic acid	2.197	[M+H]+	0.0038	0.2517	↓	0.0001	4.4714	↑
96	POS1894	8-Methylquinoline	7.69	[M+H]+	0.0015	0.1644	↓	0.0236	7.6483	↑
97	POS2001	1H-Indene-1,2(3H)-dione	2.572	[M+H]+	0.0159	1.3168	↑	0.0001	0.5893	↓
98	POS2086	Cinnamic acid	5.716	[M+H]+	0.0040	1.4981	↑	0.0005	0.5339	↓
99	POS21275	SM(d18:2/16:0)	12.543	[M+CH3OH+H]+	0.0247	0.3237	↓	0.0070	0.0138	↓
100	POS2156	6-Methoxypurine	6.123	[M+H]+	0.0003	1.9136	↑	0.0146	0.6774	↓
101	POS2344	Histidine	1.318	[M+H]+	0.0016	1.6746	↑	0.0019	0.6395	↓
102	POS2505	N-Hydroxy-1-aminonaphthalene	6.031	[M+H-H2O]+	0.0346	0.4541	↓	0.0420	2.8734	↑
103	POS2575	2,8-Dihydroxyquinoline	8.989	[M+H]+	0.0203	0.0190	↓	0.0316	28.4439	↑
104	POS2592	Carnitine	1.404	[M+H]+	0.0019	1.5256	↑	0.0014	0.5689	↓
105	POS2730	6-Methylpiperidine-2-Carboxylic Acid	5.713	[M+Na]+	0.0025	1.5013	↑	0.0003	0.5455	↓
106	POS2865	3-Methylhistidine	1.322	[M+H]+	0.0001	1.9552	↑	0.0002	0.5628	↓

107	POS3052	Arginine	1.312	[M+H]+	0.0201	1.4248	↑	0.0001	0.5299	↓
108	POS3137	Rhubafuran	9.026	[M+H]+	0.0025	0.3042	↓	0.0161	3.4475	↑
109	POS3314	Tyrosine	2.572	[M+H]+	0.0293	1.2823	↑	0.0002	0.5954	↓
110	POS3392	Phosphocholine	1.332	[M]+	0.0031	1.8470	↑	0.0123	0.6617	↓
111	POS3580	Trimethyllysine	1.31	[M+H]+	0.0002	1.8956	↑	0.0000	0.5766	↓
112	POS3643	Chrysogine, (-)-	9.759	[M+H]+	0.0024	0.1573	↓	0.0155	4.2384	↑
113	POS4168	N,N-Dimethylarginine	1.449	[M+H]+	0.0187	1.7055	↑	0.0018	0.5041	↓
114	POS4700	Tschimganidin	6.357	[M+2Na]+	0.0450	0.7273	↓	0.0233	1.5988	↑
115	POS5000	1,6-Cleve's acid	9.698	[M+H]+	0.0001	0.1057	↓	0.0198	10.6650	↑
116	POS5472	Metolachlor Morpholinone	8.702	[M+H]+	0.0010	0.0931	↓	0.0167	21.1958	↑
117	POS557	3-Azetidinecarboxylic acid	1.341	[M+H]+	0.0007	1.5458	↑	0.0013	0.6959	↓
118	POS628	Choline	1.384	[M]+	0.0279	1.5872	↑	0.0031	0.6050	↓
119	POS6868	Caffeoylcholine	8.377	[M]+	0.0019	0.4851	↓	0.0012	2.8552	↑
120	POS697	Guaiacol	5.714	[M+H-H2O]+	0.0345	1.3611	↑	0.0031	0.5779	↓
121	POS6978	4,4'-Dimethoxychalcone	11.803	[M+H]+	0.0002	0.1708	↓	0.0151	5.8041	↑
122	POS7282	3-Hydroxyhexanoylcarnitine	6.248	[M+H-H2O]+	0.0002	2.8355	↑	0.0022	0.4252	↓
123	POS7305	Tanshinone	10.541	[M+H]+	0.0081	0.4868	↓	0.0351	2.1110	↑
124	POS7517	Enterolactone	12.17	[M+H-H2O]+	0.0017	0.2611	↓	0.0321	4.9260	↑
125	POS792	3,4-Diaminopyridine	1.318	[M+NH4]+	0.0046	1.6236	↑	0.0228	0.6733	↓
126	POS8143	2-(6,8-dimethyl-4-oxochromen-2-yl)benzoic acid	10.864	[M+H]+	0.0117	0.1637	↓	0.0431	4.3578	↑
127	POS8158	Isoleucyltyrosine	7.907	[M+H-H2O]+	0.0330	0.2034	↓	0.0451	2.4374	↑
128	POS8558	N2-gamma-L-Glutamyl-L-arginine	1.377	[M+H]+	0.0098	2.5617	↑	0.0041	0.3098	↓

129	POS8563	3-hydroxyoctanoyl carnitine	9.756	[M+Na]+	0.0004	4.3369	↑	0.0096	0.4509	↓
130	POS910	2,5-Piperazinedione	1.754	[M+H]+	0.0145	1.8202	↑	0.0322	0.5741	↓
131	POS9708	4,8 Dimethylnonanoyl carnitine	12.481	[M+CH3OH+H]+	0.0096	1.8761	↑	0.0106	0.5466	↓
132	POS9939	Berberine	11.544	[M]+	0.0023	0.4325	↓	0.0320	6219.0679	↑
