# Supplementary material for: Pulsatilla Powder Ameliorates Damp-Heat Diarrhea in Piglets Through the Regulation of Intestinal Mucosal Barrier and the Pentose Phosphate Pathway Involving G6PD and NOX
Source: Vet Sci. 2025 Apr 25;12(5):403. doi: 10.3390/vetsci12050403 (PMC12116046; doi:10.3390/vetsci12050403)
Supplement: Supplementary file 1 [file vetsci-12-00403-s001.zip › Supplementary file 2, Significant Metabolic Pathways.pdf]

Supplementary file 2, Significant Metabolic Pathways

			NC vs M					M vs PP				
ID	Description	SecondClass	Count	RichFactor	ImpactBC	ImpactODC	pvalue	Count	RichFactor	ImpactBC	ImpactODC	pvalue
ssc00030	Pentose phosphate pathway	Carbohydrate metabolism	1	1.64	0.0246	0.02	0.4281	1	1.81	0	0.02	0.4281
ssc00052	Galactose metabolism	Carbohydrate metabolism	1	1.28	0.0011	0.0435	0.5108	1	1.42	0.0011	0.0435	0.5108
ssc00053	Ascorbate and aldarate metabolism	Carbohydrate metabolism	1	1.04	0	0	0.0109	4	4.57	0.147	0.1538	0.0109
ssc00120	Primary bile acid biosynthesis	Lipid metabolism	2	2.51	0.0586	0.0213	0.0347	3	4.16	0.0586	0.1489	0.0347
ssc00130	Ubiquinone and other terpenoid-quinone biosynthesis	Metabolism of cofactors and vitamins	1	0.83	0	0.0666	0.6693	1	0.92	0	0.0666	0.6693
ssc00140	Steroid hormone biosynthesis	Lipid metabolism	2	1.19	0.0047	0.011	0.1935	3	1.98	0.0092	0.055	0.1935
ssc00220	Arginine biosynthesis	Amino acid metabolism	4	10.28	0.4445	0.3888	0.0004	4	11.34	0.2518	0.3333	0.0004
ssc00230	Purine metabolism	Nucleotide metabolism	4	2.34	0.1117	0.0797	0.0042	6	3.87	0.1121	0.1239	0.0042
ssc00232	Caffeine metabolism	Biosynthesis of other secondary metabolites	1	2.69	0	0	0.0440	2	5.93	0	0	0.0440
ssc00240	Pyrimidine metabolism	Nucleotide metabolism	1	0.92	0.0171	0.0294	0.0162	4	4.07	0.0852	0.0441	0.0162
ssc00250	Alanine, aspartate and glutamate metabolism	Amino acid metabolism	3	6.33	0.335	0.2105	0.0008	4	9.31	0.3553	0.2367	0.0008
ssc00260	Glycine, serine and threonine metabolism	Amino acid metabolism	5	6.16	0.091	0.0566	0.0059	4	5.43	0.2507	0.1887	0.0059
ssc00270	Cysteine and methionine metabolism	Amino acid metabolism	2	1.76	0.0557	0.0238	0.0188	4	3.89	0.0557	0.0714	0.0188
ssc00310	Lysine degradation	Amino acid metabolism	3	3.17	0.002	0.0968	0.2119	2	2.33	0	0.0645	0.2119
ssc00330	Arginine and proline metabolism	Amino acid metabolism	7	5.99	0.2934	0.2618	0.0208	4	3.78	0.1525	0.1904	0.0208
ssc00340	Histidine metabolism	Amino acid metabolism	5	6.29	0.5199	0.4375	0.0055	4	5.55	0.32	0.3126	0.0055
ssc00350	Tyrosine metabolism	Amino acid metabolism	2	1.52	0.1155	0.1021	0.7038	1	0.84	0.1155	0.1021	0.7038
ssc00360	Phenylalanine metabolism	Amino acid metabolism	2	2.41	0.2963	0.25	0.5332	1	1.33	0	0	0.5332

ssc00380	Tryptophan metabolism	Amino acid metabolism	2	1.42	0	0	0.7262	1	0.79	0	0	0.7262
ssc00400	Phenylalanine, tyrosine and tryptophan biosynthesis	Amino acid metabolism	4	6.75	0.9999	0.6	0.0159	3	5.59	0.4999	0.2	0.0159
ssc00410	beta-Alanine metabolism	Metabolism of other amino acids	4	7.39	0	0.0345	3.43E-0.5	7	14.26	0.0549	0.1379	3.43E-0.7
ssc00470	D-Amino acid metabolism	Metabolism of other amino acids	5	4.28	0	0	0.0039	5	4.72	0	0.1111	0.0039
ssc00480	Glutathione metabolism	Metabolism of other amino acids	2	3.11	0.0159	0.054	0.1147	2	3.43	0.0159	0.027	0.1147
ssc00500	Starch and sucrose metabolism	Carbohydrate metabolism	1	1.6	0.0218	0.037	0.4370	1	1.76	0.0218	0.037	0.4370
ssc00520	Amino sugar and nucleotide sugar metabolism	Carbohydrate metabolism	1	0.5	0.0738	0.0606	0.2710	3	1.66	0.1171	0.106	0.2710
ssc00562	Inositol phosphate metabolism	Carbohydrate metabolism	1	1.26	0.0334	0.025	0.1619	2	2.77	0.0334	0.05	0.1619
ssc00564	Glycerophospholipid metabolism	Lipid metabolism	3	3.17	0.0912	0.0943	0.0102	4	4.66	0.0992	0.0943	0.0102
ssc00590	Arachidonic acid metabolism	Lipid metabolism	3	2.24	0	0	0.7084	1	0.83	0	0	0.7084
ssc00592	alpha-Linolenic acid metabolism	Lipid metabolism	2	2.69	0	0	0.1457	2	2.96	0	0	0.1457
ssc00600	Sphingolipid metabolism	Lipid metabolism	2	4.38	0.0654	0.0834	0.0637	2	4.83	0	0.0556	0.0637
ssc00650	Butanoate metabolism	Carbohydrate metabolism	1	1.26	0.0228	0.0476	0.5183	1	1.39	0.0228	0.0476	0.5183
ssc00730	Thiamine metabolism	Metabolism of cofactors and vitamins	1	1.91	0	0	0.3818	1	2.1	0	0	0.3818
ssc00760	Nicotinate and nicotinamide metabolism	Metabolism of cofactors and vitamins	3	3.22	0	0.1154	0.0515	3	3.56	0	0.1154	0.0515
ssc00770	Pantothenate and CoA biosynthesis	Metabolism of cofactors and vitamins	2	3.94	0.0211	0.0909	0.0010	4	8.69	0.0957	0.1817	0.0010
ssc00860	Porphyrin metabolism	Metabolism of cofactors and vitamins	2	0.8	0	0	0.6697	2	0.88	0.175	0.1071	0.6697
ssc00970	Aminoacyl-tRNA biosynthesis	Translation	5	5.68	0	0.1726	0.0001	6	7.52	0	0.2417	0.0001
ssc00980	Metabolism of xenobiotics by cytochrome P450	Xenobiotics biodegradation and metabolism	1	0.49	0	0	0.8500	1	0.54	0	0	0.8500
ssc01100	Metabolic pathways	Global and overview maps	57	1.11	0.0478	0.0521	0.0001	60	1.28	0.07	0.0606	0.0001
ssc01200	Carbon metabolism	Global and overview maps	3	1.56	0.0073	0.019	0.0297	5	2.86	0.0379	0.0666	0.0297
ssc01210	2-Oxocarboxylic acid metabolism	Global and overview maps	4	1.76	0	0.0477	0.1490	4	1.95	0	0.0953	0.1490

ssc01230	Biosynthesis of amino acids	Global and overview maps	8	3.69	0.0146	0.077	0.0001	9	4.58	0.0523	0.1026	0.0001
ssc01232	Nucleotide metabolism	Global and overview maps	5	5.09	0.1112	0.0877	2.37E-0.5	7	7.87	0.1628	0.1228	2.4E-0.5
ssc01240	Biosynthesis of cofactors	Global and overview maps	9	1.62	0.0125	0.0465	0.0012	13	2.58	0.0688	0.062	0.0012
ssc01250	Biosynthesis of nucleotide sugars	Global and overview maps	1	0.3	0.0464	0.0233	0.5992	3	0.98	0.0907	0.0698	0.5992
